# A Comparative Study between the Postoperative Complications of Stripping Esophagectomy and Classic (Orringer's Technique) Esophagectomy

**DOI:** 10.1055/s-0041-1736666

**Published:** 2022-02-01

**Authors:** Mojtaba Ahmadinejad, Mozaffar Hashemi, Abbas Tabatabai

**Affiliations:** 1 Department of General Surgery, Faculty of Medicine, Úlborz University of Medical Sciences, Karaj, Iran; 2 Department of General Surgery, Faculty of Medicine, Isfahan University of Medical Sciences, Isfahan, Iran

**Keywords:** stripping, nasogastric tube, transhiatal esophagectomy, Orringer, stripping, esophageal carcinoma

## Abstract

Recent studies have suggested that morbidity and mortality rate of transhiatal esophagectomy is comparable to that of thoracotomy, calling the need for the modifications in the surgical procedures. Our methodology includes stripping of esophagus by nasogastric tube to reduce the manipulation of thoracic cavity and associated complications. We also present the comparison between the stripping and classic (Orringer's technique) esophagectomy.

Patients presenting esophageal carcinoma from 2015 to 2017 were the target of this study. Patients undergoing esophagectomy were randomized to have classic or stripping esophagectomy. Operating time, manipulation time, blood losses during the surgery, duration of hospitalization, volume intake, hypotension time, arrhythmia, and transfusion were the recorded parameters. Complications, such as anastomotic leak, cardiac effects, and morbidity, were also studied. Seventy patients were referred for transhiatal esophagectomy for esophageal carcinoma at the Al Zahra Hospital. Mean ages of patients in the stripping and Orringer group were 64.00 ± 10.57 and 57.42 ± 12.20 years, respectively. Manipulation time, operating time, blood loss during the surgery, and transfusion were statistically significant variables between the two groups. Although volume intake and duration of hospitalization were not significantly different parameters, however, betterment in the outcomes was evident. Substantial decrease in overall complications via stripping method was obtained, hence can be suggested as an effective alternative, to remove the need of thoracotomy, for transhiatal esophagectomy.

Esophageal cancer contributes as the eight most frequent cancers, reported globally. Histologically, it can be either seen as squamous cell carcinoma, dominant one, or adenocarcinoma, each associated with differential epidemiology risks and consequences. It is mostly common in males and Caucasians, while adenocarcinoma is chiefly reported in Chinese population. Factors that primarily contribute to the incidence of this cancer include obesity, bacterial and viral infections, smoking, alcohol, gastroesophageal reflux disease, Barrett's esophagus, and side effects of certain drugs. 1

Depending on the severity of the disease, several treatment options are available. Premalignant treatment is successfully provided using mucosal resection and radiofrequency ablation, 2 whereas localized cancer is operated by esophagectomy. 3

Several surgical options withstand in case of benign and malignant esophageal lesions. 4 Benign or malignant condition of the lesion, the extent of the lesion, location of the tumor, and the presence of complications are some of the factors which determine the type of surgical procedure required, 5 nonetheless, esophagectomy is usually recommended for the patients with benign conditions. 6 These surgical procedures include transthoracic esophageal resection by either right or left thoracotomy and transhiatal esophageal resection (Orringer's esophagectomy) without thoracotomy. Nonetheless, low-to-no difference in survival rate has been noted between either of these procedures. 7 Transhiatal method includes removal of greater number of metastasized lymph nodes, which is operated for adenocarcinoma-type esophageal cancer. 8 Recent studies suggest transhiatal esophagectomy has morbidity and mortality rates comparable to thoracotomy esophagectomy which cause this surgical procedure as an alternative to traditional transthoracic esophagectomy. 9 10 11 Complications such as hemorrhage (due to the damage of the azygos vein, aortic esophageal artery, or thoracic aorta), anastomotic leak, mediastinitis, pulmonary complications, arrhythmia, and anastomotic stricture may occur during or after the procedure with esophageal resection and reconstruction. 12 13 14 Dissection of the esophagus from the posterior mediastinum can be hemodynamically challenging. 15 Less manipulation and reducing operative-chest involvement during procedure can, however, reduce morbidity and mortality in this surgical procedure.

This study provides an alternative technique for transhiatal esophagectomy and provides comparative analysis.

## Materials and Methods

### Subject Recruitment

Patients presenting esophageal carcinoma (benign) were enrolled in this study. Patients were randomly assigned to undergo classic transhiatal (Orringer's technique) or stripping esophagectomy (eversion esophagectomy). G Power software is used to estimate sample size (Faul et al 16 ).

### Operation Technique

A nasogastric tube was fixed in stomach before operation. Abdominal and cervical neck incision was performed in transhiatal esophagectomy without opening thoracic cavity. To it, stomach was entirely immobilized for the procedure. Lymph nodes surrounding the distal part of the esophagus, the gastric cardia, omentum, and the left gastric artery were removed. Blunt method was exploited to cut the intrathoracic part of the esophagus, distancing it from adjacent thoracic structures.

To accomplish this procedure, the surgeon opened the diaphragmatic hiatus and mobilized the esophagus by dissecting up into the thoracic cavity. The cervical component of the operation involved opening the neck followed by the lateral retraction of sternocleidomastoid.

**Fig. 1:**
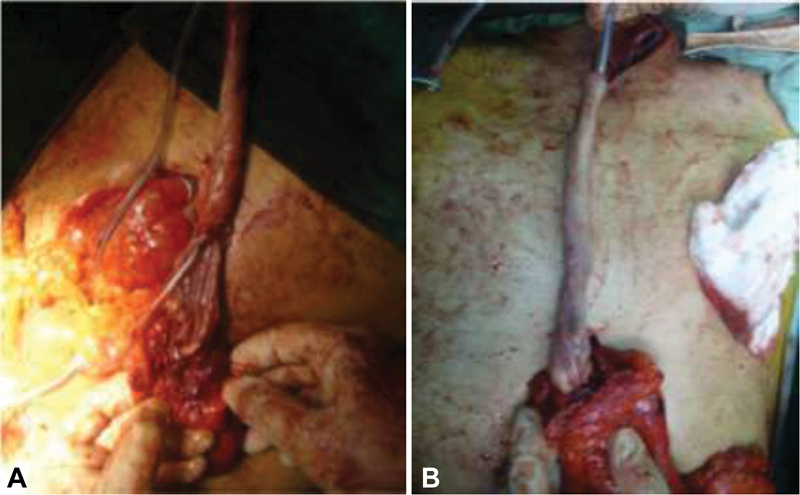
The esophagus view ( **A** ) before and ( **B** ) after stripping.

The part of the esophagus in the neck was isolated and dissected away from the flanking trachea. In our procedure the esophagus was then partially dissected in the neck. Nasogastric tube fixation was made free from the nose and brought out of the field approximately 30 cm then esophagus was tied to the nasogastric tube from the distal part. Partial incision of neck esophagus was made complete, and from a small incision in the esophagogastric junction, the tip of the nasogastric tube was pulled ( Fig. 1 ).

After division of the upper part of the stomach, along with the esophagus, it was sent for histological examination to pathology laboratory. To establish gastrointestinal continuity, remaining part of the stomach was reconnected with the tube, passing it through the posterior mediastinum while connecting the cervical region of the esophagus to the stomach passing the tube up through the posterior mediastinum and by manual stitches using 3–0 Vicryl. All the operations were performed by the same surgeon.

The time of the operation, manipulation time, blood loss during the surgery, duration of hospitalization, volume intake, hypotension, and transfusion were noted. Other complications, such as anastomotic leak, cardiac effects, and mortality, were also under consideration. After the surgery, patients were assessed for anastomotic leakage by a meglumine diatrizoate (Gastrografin) contrast study performed on day 7, postoperatively. Anastomotic leakage was diagnosed based on clinical and radiological confirmations.

Patients were followed-up every 2 weeks for 2 months and monthly thereafter for 1 year, then at 3-monthly intervals after their discharge from the hospital. If symptoms of dysphagia returned, endoscopic and barium swallow examinations were performed. Diagnosis regarding benign anastomotic narrowing was done during endoscopy; passage of a 10-mm diameter flexible endoscope. Histological evidences showed malignant narrowing.

For our analysis, development of the benign stricture, patients' death in the hospital, anastomotic leakage, or recurrence of malignancy were excluded. G Power software is used to estimate sample size. 16

### Power Analysis

We used independent *t* -test and chi-square test to compare quantitative and qualitative complications between stripping and classic esophagectomy, respectively.

### Power Analysis for Independent *t* -Test

**Fig. 2:**
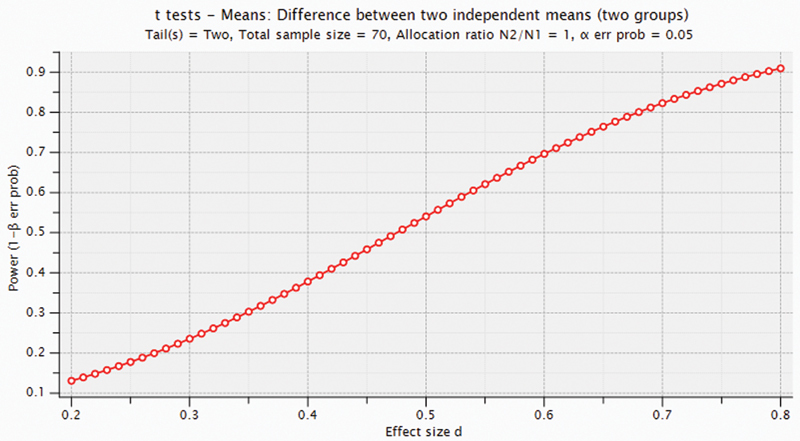
Achieved power given different effect sizes at significant level ( *α* = 0.05) and sample size 70 (35 per each group).

As we can see in Fig. 2 , *t* -test can find moderate to large differences (0.6 < *d* < 0.8) with a reasonable power (1– *β* > 0.7). 17

### Power Analysis for Chi-Square Test

**Fig. 3:**
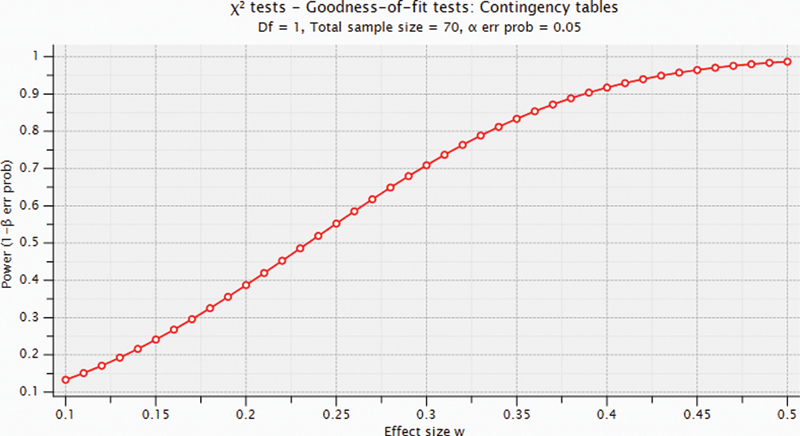
Achieved power given different effect sizes at significant level ( *α* = 0.05) and total sample size 70 (35 per each group).

As we can see in Fig. 3 , chi-square test can find moderate to large differences (0.3 < *d* < 0.5) with a reasonable power (1– *β* > 0.7).

### Statistical Analysis

Statistical analysis among the two groups was conducted using Student's *t* -test and the chi-square test. A *p* -value of 0.05 was regarded as significant. Data were analyzed using SPSS 15.0 (SPSS Inc., Chicago, IL) software.

### Ethical Consideration

Study protocols were approved by the Ethical Review Board of Isfahan University of Medical Sciences. Written informed consent was endorsed either by all the individual participants of the study or their parents/guardians.

## Result

In the period of our study, altogether 70 patients presenting esophageal carcinoma were suggested for transhiatal esophagectomy at the Al Zahra Hospital. Mean ages of the participating patients in the stripping and Orringer groups were 64.00 ± 10.57 and 57.42 ± 12.20 years, respectively. Manipulation and operating time, blood loss during the surgery, and transfusion were the statistically significant variables among the two groups ( Table 1 ). Anastomotic leakage was reported in 7 patients in the stripping group and in 5 patients in the Orringer group, which was not statistically significant, *p* = 0.75. Additionally, anastomotic stricture in these two groups was reported in 4 patients, respectively, *p* = 1.0 ( Table 1 ).

**Table 1:** Demographic data, operative, and postoperative data comparison between two groups

Age (y) (mean ± SEM)	Stripping ( *n* = 35) 64.00 ± 10.57	Orringer ( *n* = 35) 57.42 ± 12.20	*p*
Sex (%)			
Male	25 (71.5)	20 (57.1)	0.318
Female	10 (28.6)	15 (42.9)	
Manipulation time (min ± SEM)	5.42 ± 1.53	7.74 ± 2.27	0.00
Operating time (min ± SEM)	99.71 ± 20.61	112.14 ± 12.14	0.003
Blood loss (mL ± SEM)	442.85 ± 109.98	530.00 ± 121.99	0.003
Volume intake (L ± SEM)	1.43 ± 0.22	1.60 ± 0.35	0.85
Duration of hospitalization	10.51 ± 3.99	12.05 ± 5.83	0.20
Transfusion (%)			
Yes	0	6 (17.1)	0.025
No	35	29 (81.9)	
Hypotension during surgery			
Yes	15 (42.9)	22 (62.9)	0.15
No	20 (57.1)	13 (37.1)	
Reoperation	6 (17.1)	10 (28.6)	0.39
Anastomotic leaks (%)			
Yes	7 (20)	5 (14.3)	
No	28 (80)	30 (85.7)	0.75
Anastomotic stricture(%)			
Yes	4 (11.4)	4 (11.4)	1.00
No	31(88.6)	31 (88.6)	
Cardiac complication (%)			
Yes	13 (37)	16 (45)	0.62
No	22 (63)	19 (55)	
30-day mortality (%)			
Yes	3 (8.57)	3 (8.57)	1.00
No	32 (91.43)	32 (91.43)	

Abbreviation: SEM, standard error of the mean.

Although volume intake and duration of hospitalization were not significant, they implicated betterment in the outcomes. Note that 64.5% of patients with Orringer striping surgery suffered from hypotension, 11.5% of patients needed transfusion during surgery (3.3% in the first group and 19.4% in the second group). Of 31 patients with arrhythmia, 9 patients (29%) had premature atrial contraction (PAC) (1), 5 patients (16.1%) sinus bradycardia, and 16 patients (54.9%) presented PAC-premature ventricular contraction (PVC) (2). Mean time of manipulation in the three types of arrhythmia was not significantly different ( *p* = 0.36). Five patients with preoperative PAC-related arrhythmias, continued to have them perioperatively. The most common type of arrhythmias in classic striping surgery was bradycardia (40%), whereas in classical surgery it was PAC-PVC (66.2%) ( *p* = 0.03). The mean hypotension time was 4.28 ± 1.06 minutes (max 70 and at least 30 minutes), in the first group it was 3.5 ± 0.7 minutes and in the second group 4.68 ± 1 minutes. After surgery, 10.16% of patients had arrhythmia in first 24 hours, 47.5% of patients had complications and 8.57% of the patients died within the first 30 days in both the groups.

In the acquired pathology, it was found that 62.3% patients with squamous cell carcinoma, 36.1% had adenocarcinoma, whereas 1.6% of those were inflicted with squamous adenocarcinoma. To examine the relationship between existing variables with the development of arrhythmias, the following items were discerned statistically significant: need for transfusion, the type of pathology of the tumor, the presence of arrhythmia before the surgery, forced expiratory volume in 1 second (FEV1) of less than 2 L, and the amount of fluid received ( Tables 2 and 3 ).

**Table 2:** Relationship between arrhythmias and hypotension with qualitative variables

Variable hypotonia arrhythmia	Hypotonia		*p* -Value	Arrhythmia		*p* -Value
	No	Yes		No	Yes	
Cigarette	29.6%	29.4%	0.98	32.3%	26.7%	0.63
Classical stripping method	53.4% 35.5%	46.7% 64.5%	0.16	66.7% 35.5%	33.3% 64.5%	0.015
Transfusion	2.7%	17.6%	0.12	10%	23.3%	0.005
Hypotension during manipulation				45.2%	66.7%	0.09
Complications	33.3%	58.8%	0.04	45.2%	50%	0.7
Mortality	7.7%	11.8%	0.68	10%	10%	1
SCC adenocarcinoma pathology	42.1% 45.5%	57.9% 59.4%	0.52	48.4% 51.6%	80% 20%	0.02
Arrhythmia before manipulation	11.1%	5.9%	68%	0%	16.7%	0.02
Hypotension before manipulation	7.4%	8.8%	1	3.2%	13.3%	19%
Weight loss 20%	22.2%	33.3%	34%	29%	27.6%	9%
Reoperation	14.8%	28.2%	0.04	29%	26.7%	83%
Arrhythmia during manipulation	37%	58.8%	0.09%			
Albumin < 3.5	37%	66.7%	68%	61.3%	67.9%	59%
FEV1>2	56.8%	43.2%	0.015	64.9%	35.1%	0.006

Abbreviations: FEV1, forced expiratory volume in 1 second; SCC, squamous cell carcinoma.

**Table 3:** Arrhythmia and hypotension relation with quantitative variables

Arrhythmia and variable hypotension	Arrhythmia		*p* -Value	Hypotension		*p* -Value
	Yes	No		Yes	No	
FEV1	2.02 ± 0.45	2.06 ± 0.39	0.78	1.93 ± 0.28	2.17 ± 0.44	0.03
Manipulation time	6.9 ± 2.29	6.67 ± 2.5	0.71	7.64 ± 2.42	5.7 ± 1.87	0.001
Operating time	110.33 ± 21.65	102.41 ± 12.44	0.08	105.73 ± 13.3	107.03 ± 22.6	0.78
Blood loss	541.66 ± 18.198	472.58 ± 104.75	0.07	541.17 ± 172.53	462.96 ± 105.24	0.04
Systolic blood pressure	12.66 ± 1.09	12.51 ± 0.76	0.53	12.91 ± 0.86	12.18 ± 0.87	0.002
Diastolic blood pressure	8.36 ± 0.76	8.29 ± 0.82	0.7	8.58 ± 0.65	8 ± 0.83	0.03
Volume intake	1.69 ± 0.42	1.47 ± 0.19	0.01	1.65 ± 0.38	1.49 ± 0.27	0.06
Duration of hospitalization	13.7 ± 10.73	11.3 ± 3.97	0.25	13.88 ± 9.69	10.69 ± 5.07	0.13

Abbreviation: FEV1, forced expiratory volume in 1 second.

In relation to the existing variables with hypotension, duration of manipulation, amount of bleeding, systolic and diastolic blood pressure before manipulation, and FEV1 less than 2 L were correlated. In patients with arrhythmia, the complications and postoperative mortality were not significantly different from that of the control group, but in patients with hypotension, the overall complications and need for reoperation were significantly more than the control group. In all treated patients, the study was considered for coagulation disorder where international normalized ratio was normal in both the groups. No patients demonstrated extensive bleeding perioperatively, therefore there was no need for a keratectomy. The relationship between the variables with the arrhythmia surgery, duration of mediastinal surgery and operation, duration of hypotension during mediastinal surgery, and blood transfusion rates were significantly lower in the striping method.

## Discussion

Transhiatal esophagectomy is recommended for adenocarcinoma-type esophageal cancer at lower chest and cardia. However, several complications are reported in conventional method. Techniques to overcome these adverse effects include minimally invasive esophagectomy, anti-inflammatory drugs, fluid management during the surgery, and postoperative nasogastric decompression. 18 In a recent case report, esophagus was stripped due to the adhesion in the thoracic cavity in patient with esophageal carcinoma of squamous cell type. 19

Rajan et al 20 described the use of vein stripper to extract esophagus, eversion stripping, the esophagus, at the same time excising the tumor from the larynx, pharynx, and esophagus, with a significant reduction in morbidity compared with the Akiyama et al study. 21

We have successfully exploited nasogastric tube for extraction of the esophagus with a comparable reduction in morbidity. Further optimizing of the reconstructive procedure is likely to provide reduction in mortality and morbidity rates, shorter hospital stay, and rapid return to successful feeding. 22 23 A study has been reported stating insecurity of nasogastric tube, particularly, for proximal esophagus stripping hence requiring vein stripping for the purpose. 24

Postoperative complications are very important factor in choosing surgical method. In the present study, cardiac complications were one of the common complications of procedures (37%) and is comparable with previous reports. 25

In a recent survey by Orringer et al, 26 it was shown the mean blood loss was respectively 677 mL in surgeries during 1976 to 1998 and 368 mL during 1998 to 2006. In our study, the mean blood loss was 442.85 ± 109/98 mL which is significantly lower than comparable studies.

The overall anastomotic leak rate after cervical esophagogastric anastomosis has been reported 12% by Orringer et al, although in this study by stripping method anastomotic leak was 20%. The anastomotic stricture was same in the two groups of our study (11.4%); however, it is comparable to 42% that has been reported by Honkoop et al. 27

Blunt finger dissection has been reported to produce critical complications and morbidity. 28 In previous studies, 30 days mortality was from 5 to 11 to 15%, 29 30 whereas 8.57% in the present study. Decreased mortality rate in the present study probably reflects less manipulation and avoiding operative chest complications during our procedure.

Despite overall decrease in postoperative complication achieved by nasogastric stripping, comparison with other minimally invasive techniques via laparoscopy or thoracoscopy, Ivor Lewis and Mckeown esophagectomy, can provide a decisive answer. 31 Further reduction in complications and mortality rate are likely to be achieved by integrating these methods. 32 33 34
